# Effects of mobile-assisted reading materials on children’s L1 lexical development

**DOI:** 10.3389/fpsyg.2023.1144427

**Published:** 2023-06-08

**Authors:** Xueli Liu, Chuanbin Ni, Yan Liu

**Affiliations:** ^1^School of Foreign Languages, Zhengzhou University of Light Industry, Zhengzhou, China; ^2^School of Foreign Languages and Cultures, Nanjing Normal University, Nanjing, China; ^3^School of Foreign Studies, Nanjing University of Posts and Telecommunications, Nanjing, China

**Keywords:** Chinese children, learning effectiveness, primary school students, L1 lexical development, mobile-assisted reading materials

## Abstract

Despite the wide and extensive use of mobile-assisted devices, the effectiveness of children’s L1 learning with these mobile-assisted technologies has been less discussed. This study aims to explore the effects of mobile-assisted reading materials on Chinese children’s L1 vocabulary learning. We adopted a longitudinal and quasi-experimental design consisting of an experiment group using the mobile-assisted materials and a control group using the traditional paper materials, and took children’s lexical development as indexed by assessing the parameter, lexical diversity, in different testing times. The results showed that (1) children’s L1 vocabulary learning effectiveness of using mobile-assisted materials is as similar as that of using conventional paper materials in general, and (2) the changing patterns of children’s L1 lexical development using mobile-assisted materials in different testing times are various. Specifically speaking, (a) in the posttest 1 (the first month), compared with the traditional paper reading materials, the mobile-assisted reading materials have a facilitating effect on the primary school students’ L1 vocabulary learning; (b) in the posttest 2 (the second month), children’s vocabulary learning effectiveness is inhibited by the mobile-assisted reading materials; (c) in the delayed posttest (the fourth month), there is no difference in the learning effectiveness by these two different kinds of learning materials and the lexical diversity increases slowly but steadily. We analyzed the results from research-design factors and learner-related factors, hoping to shed light on children’s mobile-assisted language learning research.

## Introduction

With the advent of the information age, mobile-assisted technologies have become widely available and advocated in the field of education, and this general trend was catalyzed by the COVID-19 global pandemic. Traditional teaching was disrupted and the vast majority of educators were forced to implement some mobile-assisted devices into the established teaching methodologies in this critical and transformed phase (Malkus et al., 2020). In China, where Ministry of Education launched an initiative entitled “ensuring learning undisrupted when classes are disrupted,” numerous mobile-assisted teaching platforms and intelligent learning apps have been developed to meet this urgent requirement.

Although there are various functions for mobile-assisted language learning, it is still controversial whether learning with the mobile-assisted tools outweighed the traditional way in the classroom. A lot of discussions were made to explore this issue. In general, some researchers found that learners’ mobile-assisted language learning outcome is not so effective as traditional classroom instruction (Nielson, 2011; Handal et al., 2013; Gaudreau et al., 2014; Xodabande and Atai, 2020), but most scolars in mobile-assisted language learning (MALL) insisted that the implementations can generate positive results and facilitate learners’ language learning (e.g., Hulstijn and Laufer, 2001; Burston, 2014; Sung et al., 2015; Mahdi, 2017; Lin and Lin, 2019). However, in past research, learning with mobile-assisted tools was taken as an additional intervention, and the extra learning time is beneficial to learners, so the learning effectiveness is undoubtedly positive. In light of this consideration, the question whether this effective learning is caused by mobile-assisted technologies or by the extra learning time is still to be discussed.

In addition, most previous studies examined the effectiveness of mobile-assisted technologies by exploring learners’ language skills, such as listening (Chen and Chang, 2011; Hwang and Chen, 2013; Jia and Hew, 2019), reading (Chang and Hsu, 2011; Wang, 2017), writing (Eubanks et al., 2018; Ngui et al., 2020; Rahimi and Fathi, 2021; Min and Hashim, 2022) and speaking (Sun et al., 2017; Maulina et al., 2021; Tarigan et al., 2021), and language sub-skills, such as grammar (Chu et al., 2019), pronunciation (Wongsuriya, 2020), and vocabulary (Çakmak and Erçetin, 2018; Rassaei, 2020; Teng, 2020; Xodabande and Atai, 2020; Hao et al., 2021; Yang et al., 2021; Yu and Trainin, 2021; Zou et al., 2021; Guo et al., 2022; Lei et al., 2022; Li and Hafner, 2022; Rahmani et al., 2022; Xodabande et al., 2022). As for language sub-skills, the exploration of vocabulary learning and teaching, in comparison with studies on grammar or pronunciation, has attracted more attention and has been a relatively successful research area in MALL (Duman et al., 2015). Although previous literature has highlighted language vocabulary learning in technology-assisted settings (Lin and Lin, 2019), numerous studies have focused on the L2 vocabulary change of college students in the field of SLA (especially English vocabulary learning; Vorobel and Kim, 2012), yet young learners’ L1 lexical development is under-researched (Turan and Akdag-Cimen, 2020; Lei et al., 2022).

Chinese children often enroll in the elementary school at the age of six, and it is at this time that they begin to learn Chinese characters and improve their reading skills systematically. Under the guidance of their teachers, these primary school students learn the new characters and words by reading some paper materials. According to the New Chinese Curriculum Standard made by Ministry of Education of the People’s Republic of China, students in grades 1–2 (ages 6 to 8) have to master 1,600 Chinese characters. Following that, these students can continue their learning with the aid of Pinyin and expand their vocabulary by reading extra-curricular paper books. With the prevalence of the mobile-assisted devices in language learning, some teachers and scholars began to give students some e-books to read. However, it is still unknown whether the reading format has an impact on children’s learning effectiveness. To provide more evidence on the mobile-assisted learning effectiveness for the young learners, the present study is aimed at exploring these Chinese elementary students’ L1 lexical development with different learning formats, and is expected to shed light on children’s mobile-assisted language learning research.

## Literature review

### Vocabulary learning and measurement

Vocabulary is viewed as a fundamental factor in language acquisition in general (Rahmani et al., 2022) and is deemed essential for language learners’ competency development (Clenton and Booth, 2020). In the previous studies, multiple indicators were adopted to measure and estimate learners’ knowledge of vocabulary, in which lexical richness is one of the predictors of learners’ overall language proficiency (Laufer and Nation, 1995). Read (2000) proposed that lexical richness included four parameters, i.e., lexical diversity, lexical density, lexical complexity, and lexical error, among which, lexical diversity is of the essence. According to Johansson (2008), lexical diversity is frequently described as the range or variety of various words in a text or as “phonologically-orthographical different word forms” that are indicative of the vocabulary size (Housen et al., 2008). It is usually measured by Type–Token Ratio (TTR). *Token* and *type* are two indicators used in the study of lexical diversity. These two terms are not always the same; for instance, there are three types but ten tokens in the sentence “Rose is a rose is a rose is a rose.” TTR has usually been used to measure learners’ lexical diversity; however, this kind of measurement is susceptible to the text length (Duran et al., 2004) because the overall number of words or tokens increases as the text length increases, but the number of new word types (or new lexical words) does not increase at the same rate (Nasseri and Thompson, 2021). Besides, it was discovered that TTR was not a valid or reliable way to assess children’s lexical abilities (Bernstein Ratner and Mac Whinney, 2016), as it might not correlate with how well the same children performed on standardized tests of vocabulary comprehension or production (Hess et al., 1984). As a result, the formula *Uber index = U = (log tokens) ^2^/(log tokens-log types)* was extensively used since it is “not impacted by the length of the text” (Vermeer, 2000: 67) and can assess lexical diversity much more precisely (Tweedie and Baayen, 1998).

Additionally, learners were often given a wordlist and allowed to acquire the terms on their own or with guidance. The success of their learning was then assessed using various testing tools or vocabulary measuring scales (e.g., Rahmani et al., 2022). In most cases, the vocabulary learning effectiveness was great with this intentional and explicit learning. However, in order to enlarge one’s vocabulary size, both explicit and implicit vocabulary acquisition are required (Polakova and Klimova, 2022). The implicit vocabulary acquisition involves the incidental learning, which is a kind of learning without intent or unconsciously (Eraut, 2004; Nation, 2013). Incidental vocabulary learning occurs through reading, listening, reading-while-listening, and other related activities (Teng, 2022), in which reading is an effective way to gain vocabulary incidentally (Teng, 2020). The majority of related studies in the past have explored lexical development by measuring word recognition; however, few have examined the lexical development through incidental learning from reading, particularly from the speaking-after-reading task.

### Effectiveness of mobile-assisted vocabulary learning

With the “anywhere and anytime” features, mobile-assisted technologies have been frequently adopted by students and educators, and a substantial body of previous studies have begun to shift their focus to employ mobile-assisted technologies for language learning, especially L2 vocabulary learning (see more in Lin and Lin, 2019). The findings fall into two different stands.

The first stand insisted that language learning by mobile-assisted devices was more effective than the traditional learning. Most research in this stand adopted an experiment-control group research design to examine the different learning effectiveness via mobile-assisted technologies and traditional learning tools. For instance, Thornton and Houser (2005) explored Japanese EFL college students’ vocabulary learning in a mobile-based technology classroom, and found their vocabulary learning marks were higher than that of the students learning in traditional classrooms. Corresponding to their results, Lu (2008) found that the mobile group outperformed their paper-group classmates. Wu (2015) and Liu (2016) conducted similar empirical research to explore the effectiveness of smartphones in helping EFL college students’ vocabulary learning, and the findings also showed that the learners in the mobile-based class had better performance than those in the traditional one. To investigate some more specific effectiveness, Li and Hafner (2022) examined the differences of vocabulary learning via mobile-assisted word cards and paper word cards, and found the vocabulary learning of learners either in the mobile-assisted or in the conventional classes was improved, but the degree of improvement in the mobile-assisted class was greater than that in the conventional class. In addition, except for the quasi-experiment exploration, some researchers (e.g., Azara and Nasiri, 2014; Lin and Yu, 2017; Krishnan et al., 2020; Togaibayeva et al., 2022) tried to probe learners’ attitude toward these mobile-assisted technologies to explore their effectiveness, and found that most EFL learners have positive attitudes toward mobile-assisted devices. Moreover, empirical studies taking other L2s such as Turkish (Loewen et al., 2019), Spanish (Loewen et al., 2020), and German (Salomonsson, 2020) also got similar findings. All in all, the expanding body of research indicated that mobile-assisted learning was significantly positive; however, it has to be mentioned that most studies in this line of research were conducted in short periods (Xodabande and Atai, 2020), which makes it difficult to understand the long-term impacts of mobile-assisted vocabulary learning in general (Lin and Lin, 2019).

The second stand proposed that mobile-assisted devices may produce some negative learning effects. According to *The Mobile Internet Report in 2020*,^1^ only 25.3% of learners think mobile-assisted language learning is more effective than traditional language learning. Researchers holding this view also carried out a series of experiments to verify this point; for example, Xodabande and Atai (2020) explored the effect of frequency factor on EFL learners’ academic vocabulary and found a significant decline in the academic vocabulary. In addition, Wu (2020), by doing a series of field studies, found that the results of online education during the epidemic were negative and disappointing, because parents, students, and teachers were all exhausted. Other researchers also pointed out that mobile-assisted learning may divert students’ attention by providing some irrelevant materials during the learning process (e.g., Handal et al., 2013; Gaudreau et al., 2014). Nielson (2011) also stressed that without proper support, even learners with high motivation may fail to engage in MALL-based language learning.

Taken together, there are primarily three limitations in the previous studies. First, there is ongoing debate regarding the effectiveness of mobile-assisted vocabulary learning. Second, the L1 lexical development of young learners, particularly elementary students, has received far less attention. Third, the issue whether the effectiveness of incidental mobile-assisted vocabulary learning varies on different learning stages is less discussed. This study adopts a quantitative longitudinal study and explores the different L1 lexical developing trends of Chinese primary school students in a speaking-after-reading task with either mobile-assisted reading materials or conventional paper reading materials. The following two research questions will be addressed.

RQ1: Is it more effective to use mobile-assisted materials than conventional paper materials in helping elementary students increase their L1 lexical richness?

RQ2: How does primary school students’ L1 vocabulary develop with the assistance of mobile materials in different stages?

## Meterials and methods

### Design of the study

The current study is longitudinal and quantitative in nature and uses a pretest-posttest quasi-experimental design. Since longitudinal research examines the participants over an extended period of time, it is thought to offer researchers with rich data (Lei et al., 2022). In the present experiment lasting for 4 months, participants’ quantitative data were collected via a speaking-after-reading task at four time nodes respectively, i.e., in the beginning, the 1st month, the 2nd month and the last month.

### Participants

48 primary school pupils (26 males, 22 females) were chosen, who are non-stutterers and from two intact classes in a public primary school in a metropolitan Chinese city, and their ages ranged from 7 to 8 (*M* = 7.75). The two classes were assigned as two groups at random: the experiment group (*N* = 24) were given some e-books in the format of PDF and they can read them on the mobile-assisted devices (such as iPAD, smartphone and laptop) while the control group (*N* = 24) read the traditional paper books. In consistence with moral contemplations in educational research, informed assents were gotten from the students, their parents and their Chinese teacher. All of them were told about the research objectives, procedure of data collection, and confidentiality of personal information gathered in this study.

### Procedure

The current study was conducted in the spring semester of 2022. At the beginning of this semester, students of these two intact classes were required to read one of a series of elementary reading books every month. These books including *The Old Man and the Sea*, *The Records about Insects*, *The Little Prince*, *Twenty Thousand Leagues Under the Sea*, and *Treasure Island* were translated into Chinese and published by China Translation and Publishing House, a famous publishing press in China. After reading, all of them were required to retell the plot to their classmates and their Chinese teacher.

The procedure was structured in four time nodes. In the first time node (pretest; in the beginning of this experiment), all participants were asked to retell the book *The Old Man and the Sea* in the classroom. In the second time node (posttest 1; 1 month after the pretest), participants received different treatments due to the pandemic. For the experiment group, they read the book *The Records about Insects* in the format of PDF on some mobile assisted devices (e.g., iPAD); but for the control group, students were required to read paper books. All students are required to finish this book given by their teacher within 1 month and then share the main plot verbally with their teachers and classmates by Dingding (a famous mobile-assisted teaching platform in China). In the third time node (posttest 2; 1 month after the posttest 1), the procedure is identical to that used in the second stage, and the book they read is *The Little Prince*. After the sharing in the posttest 2, they had to finish a questionnaire about their personal information, which was taken as a complementary method to the quasi-experiment. All of their retellings in the pretest, the posttest 1 and the posttest 2 were recorded and then transcribed by the authors. After the posttest 2 was completed, the summer break began shortly. During the summer break, participants were not informed of the delayed posttest, but they had to read the two books *Twenty Thousand Leagues Under the Sea* and *Treasure Island continually*. In the last time node (delayed posttest; 2 months after the posttest 2), all pupils were asked to choose one book they read during the summer break and share its main plot, and the procedure is similar to the posttest 1 and the posttest 2. To have a high-efficiency understanding of the research procedure, please see Table 1.

**Table 1:** Experimental procedure.

Test	Time node	Reading materials
Pretest	1st (in the beginning)	*The Old Man and the Sea*
Posttest 1	2nd (in the first month)	*The Records about Insects*
Posttest 2	3rd (in the second month)	*The Little Prince*
Delayed posttest	4th (in the fourth month)	*Twenty Thousand Leagues Under the Sea* *Treasure Island*

In addition, it is impossible to ignore parents’ bridging role in connecting their kids and teachers. To give more information about their attitudes toward children’s language learning by e-materials, 36 parents (18 males, 18 females) took part in the online interview after the experiment. The interview, each lasting about 10 mins, covered 3 questions, that is, (1) do you think the efficacy of reading an e-book is as high as reading a paper book? (2) if it is not necessary, would you rather have your kid read an e-book? (3) what are the major considerations when your kid reads an e-book? It is important to note that the first two questions were in the form of a 5-level scale, in which 5 represents a highly positive response, but 1 is a very negative response. Their Chinese teacher (a 56-year-old female), who regulated the trial, was also invited to take part in an interview to share her feelings about the effectiveness of the mobile-assisted devices.

### Data collection and analysis

Aiming to compare the lexical diversity of these primary school students before and after using the mobile-assisted reading materials, a pretest was conducted to prove the starting point of students’ vocabulary proficiency. In the current study, learners’ lexical diversity through the change of *U*-value was examined. Specifically speaking, we counted the numbers of types and tokens in the retelling text, and then calculated the *U*-value from the formula *Uber index = U = (log tokens)^2^/(log tokens-log types)*. Participants share their retelling every 4 weeks during the posttest. The sharing was audio recorded and transcribed verbatim, and then analyzed with SPSS 25.0 for descriptive statistics. To further investigate the main effect of the independent variables (i.e., group with two levels and time with four levels), a repeated ANOVA was carried out.

## Results

**Figure 1:**
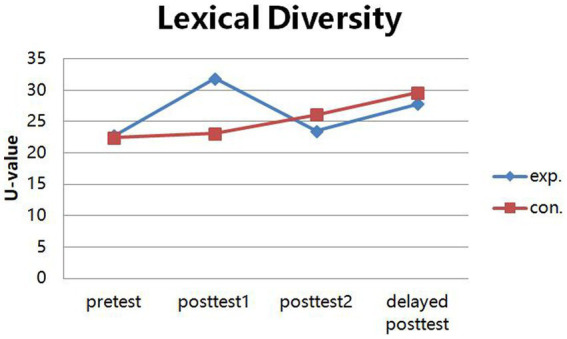
Descriptive statistics for *U*-value across time.

The results of the descriptive statistics for the lexical diversity in the pretest (Figure 1) showed that students in the two intact classes have similar *U*-values (Experiment group: *M* = 22.777, SD = 3.015; Control group: *M* = 22.409, SD = 3.786), and there is no significant difference between these two groups in *U*-value [*t* (46) = 0.373, *p* = 0.771 > 0.05], indicating that students in the two intact classes have similar lexical diversity in the beginning. In the first sharing (i.e., posttest 1), participants in the experiment group have higher *U*-value (*M* = 31.849, SD = 14.255) than the control group (*M* = 23.094, SD = 4.373). However, in the second sharing (i.e., posttest 2), the results were completely opposite, that is, participants in the control group have higher *U*-value (*M* = 26.076, SD = 6.034) than the experiment group (*M* = 23.482, SD = 4.891). In the delayed posttest after 2 months, participants in the control group have higher *U*-value (*M* = 29.587, SD = 6.155) than the experiment group (*M* = 27.835, SD = 6.554). In addition, it can be seen that *U*-value in the control group increased gradually with time, but fluctuated in the experiment group; specifically speaking, in the experiment group, it was the highest in the posttest 1, decreased in the posttest 2, but then it increased slightly in the delayed posttest after 2 months. Figure 1 provides visual representation of the changes in lexical diversity over time. This plot is helpful in observing the changing patterns of children’s lexical diversity.

In order to further analyze the changes in children’s lexical diversity over time for statistical differences, a repeated ANOVA was conducted. As it is shown in Table 2, time has a main effect [*F*(3, 44) = 13.458, *p* < 0.001, *η*^2^ = 0.479], which means that there was a statistically significant change in the lexical diversity over the four testing sessions. Moreover, the results revealed a significant interaction effect between time and group [*F*(3, 44) = 4.393, *p* = 0.009 < 0.01, *η*^2^ = 0.230], which supported the fact that the experiment group and the control group experienced distinct changes in the *U*-value over time. To explore the specific difference between the two groups, the results of between-subjects effects were analyzed. The main effect of group was not statistically significant, as shown in Table 3 [*F*(1, 46) = 1.059, *p* = 0.309 > 0.05, *η*^2^ = 0.023]. According to the findings, children’s vocabulary learning effectiveness with the mobile-assisted materials is not different from that with the traditional paper materials, but the process of lexical development for students using e-materials is more fluctuated and changeable.

**Table 2:** Multivariate tests.^a^

Effect		Value	*F*	Hypothesis df	Error df	Sig.	Partial eta squared
Time	Pillai’s trace	0.479	13.458^b^	3.000	44.000	0.000	0.479
	Wilks’ lambda	0.521	13.458^b^	3.000	44.000	0.000	0.479
	Hotelling’s trace	0.918	13.458^b^	3.000	44.000	0.000	0.479
	Roy’s largest root	0.918	13.458^b^	3.000	44.000	0.000	0.479
Time * Group	Pillai’s trace	0.230	4.393^b^	3.000	44.000	0.009	0.230
	Wilks’ lambda	0.770	4.393^b^	3.000	44.000	0.009	0.230
	Hotelling’s trace	0.300	4.393^b^	3.000	44.000	0.009	0.230
	Roy’s largest root	0.300	4.393^b^	3.000	44.000	0.009	0.230

^a^Design: Intercept + Group. Within Subjects Design: Time. ^b^Exact statistic.

**Table 3:** Tests of between-subjects effects.

Measure: *U*-value						
Transformed variable: average						
Source	Type III sum of squares	df	Mean square	*F*	Sig.	Partial eta squared
Intercept	128681.663	1	128681.663	1990.464	0.000	0.977
Group	68.494	1	68.494	1.059	0.309	0.023
Error	2973.857	46	64.649			

Table 4 shows a series of pairwise comparisons made to learn more about the long-term learning effects on mobile-assisted platforms. As for the control group, the results showed that there was no significant difference in the *U*-value only between the pretest and the posttest 1 (SE = 1.059, *p* = 0.524 > 05), implying that the lexical diversity increased gradually in the traditional learning. However, for the experiment group, the results showed that the differences between the pretest and the posttest 1 (SE = 2.924, *p* = 0.005 < 0.01), the pretest and the delayed posttest (SE = 1.393, *p* = 0.001), the posttest 1 and the posttest 2 (SE = 3.286, *p* = 0.018 < 0.05), as well as the posttest 2 and the delayed posttest (SE = 1.659, *p* = 0.015 < 0.05) were significant (see Table 4). These findings indicated that the incidental vocabulary learning from the mobile-assisted materials resulted in significant fluctuation over time; specifically speaking, it increased rapidly in the posttest 1 but declined fast to the baseline in the posttest 2 and then gradual improvement was made in the delayed posttest.

**Table 4:** Pairwise comparisons.

	Measure: *U*-value						
Group	(I) TIME	(J) TIME	Mean difference (I–J)	Std. error	Sig.^a^	95% confidence interval for difference^a^	
						Lower bound	Upper bound
Experiment group	Pretest	Postest 1	−9.072^*^	2.924	0.005	−15.121	−3.023
	Pretest	Postest 2	−0.704	1.178	0.556	−3.140	1.732
	Pretest	delayed posttest	−5.058^*^	1.393	0.001	−7.939	−2.177
	Postest 1	Postest 2	8.368^*^	3.286	0.018	1.570	15.165
	Postest 1	Delayed posttest	4.014	2.651	0.144	−1.469	9.497
	Postest 2	Delayed posttest	−4.354^*^	1.659	0.015	−7.785	−0.922
Control group	Pretest	Postest 1	−0.685	1.059	0.524	−2.875	1.505
	Pretest	Postest 2	−3.667^*^	1.628	0.034	−7.035	−0.300
	Pretest	Delayed posttest	−7.178^*^	1.286	0.000	−9.839	−4.518
	Postest 1	Postest 2	−2.982^*^	1.260	0.027	−5.590	−0.375
	Postest 1	Delayed posttest	−6.493^*^	1.259	0.000	−9.098	−3.888
	Postest 2	Delayed posttest	−3.511^*^	1.373	0.018	−6.351	−0.670

Based on estimated marginal means. *The mean difference is significant at the 0.05 level. ^a^Adjustment for multiple comparisons: Least Significant Difference (equivalent to no adjustments).

The descriptive results in Table 5 showed that these parents denied the high efficacy of e-materials (*M* = 2.913, SD = 0.848), and they would not allow their children to read e-books if it is not necessary (*M* = 1.889, SD = 0.98). The main factors they took into consideration are the great damage to children’s eyesight (94.4%), the bad effect on children’s attention (80.5%), and the development of bad reading habit (22.2%).

**Table 5:** Descriptive results of parental attitudes toward the e-books.

*N*	The efficacy of e-books		Acceptance of e-books		Major considerations		
	*M*	SD	*M*	SD	The great damage to children’s eyesight	The bad effect on children’s attention	The development of bad reading habit
36	2.913	0.848	1.889	0.98	94.4%	80.5%	22.2%

According to the interview results, the instructor admitted that the effectiveness of reading e-books was not as high as she had anticipated. “Since it is the first time for these pupils to read books through mobile-assisted devices, I assume they would be very interested in the material and motivated to read well, which suggests that their reading effectiveness will be higher than the efficacy of reading the traditional paper books. However, I am unable to provide these pupils with immediate feedback, and I do not have enough contact with their parents due to the low sharing density and my busy schedule. There, in my opinion, are the main causes of their low reading efficacy. But at the later stages, I made some improvements, such as recording their sharing and watching the playback in time to give some immediate comments, and encouraging and praising students who read e-books to give them more confidence to keep reading on the mobile-assisted devices.”

## Discussion

The present research explored the effects of mobile-assisted reading materials on Chinese children’s L1 incidental vocabulary learning in different learning stages. The key findings are shown in the following.

### Children’s L1 vocabulary learning effectiveness with mobile-assisted reading materials

Children’s L1 vocabulary learning effectiveness with mobile-assisted materials is as similar as that with conventional paper materials. According to the results of the repeated ANOVA (see in Table 2), the main effect of group was not statistically significant, indicating that it is not more effective for primary school students’ L1 vocabulary learning through mobile-assisted materials than that through traditional paper materials; in other words, learners’ vocabulary learning was not affected by the forms of learning materials. These findings are incongruent with previous studies which proposed learners’ mobile-assisted learning was more effective (e.g., Sung et al., 2015; Lin and Lin, 2019; Loewen et al., 2020), or less effective (e.g., Nielson, 2011; Handal et al., 2013; Gaudreau et al., 2014; Xodabande and Atai, 2020).

Different research designs may cause the incongruent results. For example, for the participants, most previous studies recruited college students as the participants and examined their L2 lexical perception via some vocabulary test scale (e.g., Klimova and Polakova, 2020); however, instead of adult college students, it is the elementary school pupils who were asked to participate in the current study. These young learners have limited skills in self-regulated learning (Tao and Xu, 2022). In addition, it is their L1 incidental vocabulary in production tasks but not L2 instructional vocabulary in perception that was explored. According to Sung et al. (2015), the effect size of using mobile-assisted technology in L2 learning approached a large level, while that in L1 learning was small. Thus, one possible explanation for the present study is that the participants are Chinese elementary students whose vocabulary size is small, so the lexical diversity in their production is limited, which may get to the “floor effect”; however, different from most previous studies, the present study explored learners’ L1 vocabulary learning, and compared with L2 vocabulary learning, they are more familiar with their L1 and skillful in using it, so it may get to the “ceiling floor.” Therefore, it became more difficult to improve the learning effectiveness of Chinese primary students’ L1 vocabulary via mobile devices.

### The changing patterns of children’s L1 lexical development with mobile-assisted materials in different stages

The L1 lexical diversity of children using mobile-assisted materials develops in this trend: with an initial rapid increase followed by a sudden decline, it makes steady progress finally. As shown in the repeated ANOVA (see in Table 1), the main effect of time was significant, indicating that learners’ lexical developing trend in different stages was different, and as the descriptive results shown (see in Figure 1), compared with the effective vocabulary learning in the traditional way, the changing pattern of primary school students’ L1 vocabulary learning by the mobile-assisted materials was more fluctuated in the first 2 months, but improve steadily at last. Specifically speaking, in the experiment group, students’ L1 lexical diversity in the first stage (i.e., posttest 1) was very high, then it decreased sharply in the second stage (i.e., posttest 2), but rose smoothly in the third stage (i.e., delayed posttest).

In the posttest 1, the high lexical diversity implies that children’s L1 vocabulary learning is facilitated by the mobile-assisted materials, which is inconsistent with the previous opinion that mobile-assisted learning is less effective. The different testing durations may cause this inconsistency. As reviewed by Sung et al. (2015), the duration of the mobile-assisted L1 learning in those limited previous studies was either very short (less than 1 week) or very long (more than 6 months), so some slight difference in effectiveness may not emerge in the short duration or just be covered in the long duration. Our findings concur with this explanation. The posttest 1 was conducted in the first month of the experiment, and the duration is neither too short nor too long. During this period, the effectiveness of learners’ learning performance emerged totally so that it was very easy to test. In addition, in contrast to paper-based learning materials, the mobile-assisted materials are more appealing and enjoyable to learners (Butarbutar, 2020; Kohnke, 2020), and using mobile devices as learning tools boosts their motivation (Polakova and Klimova, 2022). Therefore, learners’ learning motivation is high in this stage, and the high motivation makes them highly engage in the mobile-assisted learning and have a good outcome in lexical diversity.

In the posttest 2, the lexical diversity in the experiment group decreased sharply, which means students’ lexical development was hindered by the mobile-assisted materials. This finding is incongruent with most past studies insisting that mobile-assisted language learning was effective. Researchers (e.g., Yazdanmehr et al., 2021) found that college students thought mobile-assisted materials were more boring than the traditional ones. According to this finding, in the present study, one of the possible explanations is participants are less interested in this kind of learning and their motivation level decreases gradually. The ill-effects of the learner-related people (e.g., their parents) may cause the low motivation.

Young learners’ language learning on mobile-assisted devices relies heavily on the support of their parents and instructors (Tao and Xu, 2022) because they do not know how to learn in a self-regulated way. Parents can provide monitoring, affective, and technology support, and also play a bridging role in facilitating instructor-learner communication to boost children’s learning motivation (Togaibayeva et al., 2022). They have to keep in contact with instructors to seek their help and get better and quicker solutions to solve children’s problems. In this way, parents play a bridging role to encourage student-teacher interaction, particularly when instructors are unable to give direct help. However, according to the interview of the study, 85% of these parents had to go out to work and did not have enough free time to monitor and ensure their kids’ commitment to language learning. As a result, it’s hard for them to serve their monitoring role. Moreover, the attitudes of most parents toward the mobile-assisted learning were not positive (*M* = 2.913), because they worried about the distraction from over-focusing on the technology (80.5%), or the damage of electronic devices to their children’s eyesight (94.4%). It is similar to the findings of Tai and Ting (2011) and Calabrich (2016), who found that some parents thought their children were too young to have mobile-assisted devices and the overuse of mobile devices would decrease their communication skills. In this study, the parents pay much attention to their children’s use of mobile-assisted devices and keep in close touch with the instructor in the initial stage about the performance of their children, but as time went by, they reduced the contact and lessened their monitoring, for they set some rules for children to use the mobile devices and thought their children would not spend too much time on mobile devices; therefore, it seems to make sense that the lexical diversity in the posttest 1 was high but in the posttest 2 was low.

Moreover, the role of instructors is another even more common theme affecting learners’ vocabulary learning (e.g., Meirovitz et al., 2022; Liu and Lai, 2023). Instructors can encourage learning mindsets and provide language learners with the necessary support (Tao and Gao, 2022), and their reward-based scheme and feedback can also arouse learners’ motivation to study (Lu, 2008). In the present study, according to the interview, the instructor did not give immediate feedback to these students and their parents. It is explainable from the results in the posttest 1 that learners have high motivation and great interest in this novel learning mode, so they have a better performance than those in the traditional one; however, after the first online share in the posttest 1, these learners found that they could not get immediate feedback from her. As Chen’s (2021) finding, learners prefer teachers’ presence and assistance. Without the instructor’s monitoring, interaction, and immediate feedback, these students’ motivation on the mobile-assisted platform has been lessened, so in the posttest 2, their lexical diversity decreased sharply.

In the delayed posttest, the young learners’ lexical learning is not affected by the different forms of learning materials in the long run. According to the results of the pairwise comparisons (see in Table 3) and the descriptive results in Figure 1, it can be seen that there is a similar developing tendency in the delayed posttest, and the lexical diversity of two groups improved steadily. According to the instructor’s interview, she made some changes to give immediate feedback, have frequent communications with their parents, and encourage these students to keep reading, which made these elementary students’ interest in learning and motivation to learn keep great and high again, so their learning effectiveness improved greatly in the end. The teacher’s instructing role plays an important part in children’s language learning. In addition, the present study explored these young learners’ L1 lexical development in L1 environment. Except for the instructional learning in the classroom and the incidental learning by reading, the incidental learning by daily communication is also an important factor to enlarge their vocabulary size. Therefore, in the delayed posttest, the developing trends of all students in different groups are similar.

To sum up, in the first stage, with the interest in the new learning form, learners’ motivation was boosted to participate in the task actively, but due to the insufficient parental monitoring and the absence of instructors’ timely and direct involvement, these children may feel bored and cannot regulate themselves all the time to complete their tasks seriously, which has a severe impact on their motivation and interest, and then on the performance. With the increase of their cognitive abilities and learning skills, as well as the beneficial L1 learning environment, their lexical development improved slowly but steadily.

## Conclusion

This study aims to investigate the effects of mobile-assisted reading materials on Chinese children’s L1 lexical development. The results showed that these children’s L1 lexical learning effectiveness with the mobile-assisted materials is the same with those in the conventional way; however, regarding the changing pattern, in the short term, these children’s L1 lexical development by the mobile-assisted materials became more fluctuated than those by the traditional method, but in the long run, the developing tendency is consistent. We analyzed these results from research-design factors and learner-related factors to have a better and comprehensive understanding of these findings, which provided empirical evidence for the effects of mobile-assisted vocabulary learning in the short and long term.

The results of the current study have some implications for future MALL research and practice, as well as some insightful pedagogical implications for language learners, their instructors, and parents, which are elaborated below. First, as for the language learners themselves, they have to improve their self-regulation skills and motivation to engage in mobile-assisted language learning. The self-regulation skill is an important factor in autonomous learning (Guo et al., 2022), and with children’s growing up, they have to turn the *other-regulation* into the *self-regulation* to become autonomous learners. In addition, students’ potential to actively pursue learning is shown by their motivation (Dörnyei, 2019, p. 25) and it serves as a foundation for subsequent engagement (Martin, 2012, p. 305). To have a better learning effect, learners should have high motivation and turn it into action. Second, instructors should participate in the elementary students’ learning and monitor their learning behavior and results to provide immediate feedback and help. Due to their limited cognitive and self-regulated abilities, primary school students cannot carry the autonomous learning, thus instructors should provide them with self-regulation skills training (Guo et al., 2022). Moreover, e-learning on the mobile-assisted devices makes a long psychological distance between students and their instructors, so teachers should participate in the whole learning process and monitor every student’s learning to change their teaching plan at any time and improve teaching effectiveness. A proper reward incentive system should be set to increase students’ motivation and erase their boredom in engagement additionally. Third, parents, playing a bridging role, should accompany their children and keep in touch with the instructors so that a student-parent-teacher connection will be set according to the school-family partnerships (Sayer and Braun, 2020).

However, the generalization of the results is limited due to the small-scaled study and the few-timed data collection. The total mean scores may be disproportionately affected by some individual extreme scores. It gives direction to future research which can expand the experimental participants and increase the sampling points. Besides, more parameters such as lexical complexity, lexical density and lexical error should be analyzed, and peer feedback should be added to the analysis of future study to comprehensively probe the factors of language learning effectiveness.

## Data availability statement

The original contributions presented in the study are included in the article/Supplementary material, further inquiries can be directed to the corresponding author.

## Ethics statement

The studies involving human participants were reviewed and approved by Ethics Committee of Zhengzhou University of Light Industry. Written informed consent to participate in this study was provided by the participants’ legal guardian/next of kin.

## Author contributions

All authors listed have made a substantial, direct, and intellectual contribution to the work and approved it for publication.

## Funding

This paper was supported by the Education Department of Henan Province (No. 2023ZZJH367), Jiangsu Social Science Foundation (No. 21YYB007), the philosophy and social science researches in colleges and universities of Jiangsu Province (No. 2021SJA0102), and Research and Practice of Higher Education Teaching Reform in Henan Province (No. 2021SJGLX199).

## Conflict of interest

The authors declare that the research was conducted in the absence of any commercial or financial relationships that could be construed as a potential conflict of interest.

## Publisher’s note

All claims expressed in this article are solely those of the authors and do not necessarily represent those of their affiliated organizations, or those of the publisher, the editors and the reviewers. Any product that may be evaluated in this article, or claim that may be made by its manufacturer, is not guaranteed or endorsed by the publisher.

## Footnotes

^1^ http://www.100ec.cn/detail--6588961.html
