# Supplementary material for: Effects of mobile-assisted reading materials on children’s L1 lexical development
Source: Front Psychol. 2023 Jun 8;14:1144427. doi: 10.3389/fpsyg.2023.1144427 (PMC10285657; doi:10.3389/fpsyg.2023.1144427)
Supplement: Supplementary file 1 [file Data_Sheet_1.docx]

Supplementary Material

**Effects of Mobile-assisted Reading Materials on Children’s L1 Lexical development**

Xueli Liu, Yan Liu, Chuanbin Ni*

*** Correspondence:** Chuanbin Ni: nichuanbin@263.net

# Supplementary Data

| group | | U-value | | | |
| --- | --- | --- | --- | --- | --- |
| Experiment group | id | pretest | posttest1 | posttest2 | delayed posttest |
|  | 1 | 25.01642 | 69.0615 | 24.4119 | 35.34776 |
|  | 2 | 19.27976 | 37.9778 | 30.84699 | 36.08377 |
|  | 3 | 20.086 | 25.87336 | 29.59179 | 26.66377 |
|  | 4 | 24.38389 | 23.01044 | 18.10602 | 30.24209 |
|  | 5 | 30.12479 | 34.12047 | 24.05737 | 22.29875 |
|  | 6 | 22.18277 | 17.22391 | 24.76758 | 23.95223 |
|  | 7 | 18.6876 | 30.8691 | 17.99257 | 19.74104 |
|  | 8 | 22.55675 | 18.56574 | 22.04576 | 24.22608 |
|  | 9 | 24.59451 | 27.94524 | 13.60353 | 24.5264 |
|  | 10 | 22.93211 | 18.42212 | 29.92887 | 29.52701 |
|  | 11 | 17.77736 | 52.02078 | 14.28007 | 29.18415 |
|  | 12 | 22.77724 | 15.76084 | 23.85453 | 26.43273 |
|  | 13 | 25.08809 | 27.47075 | 25.4818 | 26.70314 |
|  | 14 | 17.84491 | 26.08301 | 21.04151 | 18.92055 |
|  | 15 | 24.67074 | 30.0311 | 29.39338 | 32.85524 |
|  | 16 | 24.8774 | 37.6768 | 23.91191 | 19.23021 |
|  | 17 | 19.42069 | 25.12433 | 22.07418 | 28.60956 |
|  | 18 | 20.20987 | 24.37788 | 27.80262 | 24.66006 |
|  | 19 | 26.2472 | 28.37702 | 19.83633 | 25.48654 |
|  | 20 | 22.25297 | 31.84919 | 30.01994 | 18.0427 |
|  | 21 | 25.62363 | 36.43611 | 22.67821 | 31.1094 |
|  | 22 | 21.50598 | 29.07168 | 27.76605 | 30.18623 |
|  | 23 | 24.84733 | 25.14297 | 21.80378 | 43.48548 |
|  | 24 | 23.66582 | 71.88844 | 18.26326 | 40.53022 |
| Control group | 1 | 16.91778 | 26.10027 | 38.79902 | 31.71892 |
|  | 2 | 18.33703 | 24.21514 | 35.16712 | 39.39241 |
|  | 3 | 17.46563 | 19.88682 | 26.54951 | 19.84467 |
|  | 4 | 17.36367 | 20.81373 | 24.66241 | 22.40569 |
|  | 5 | 21.99976 | 27.88561 | 24.45865 | 28.5031 |
|  | 6 | 24.31286 | 17.97888 | 19.01899 | 18.24871 |
|  | 7 | 23.01053 | 19.57977 | 34.93289 | 32.0162 |
|  | 8 | 18.98629 | 20.29353 | 22.03177 | 20.16576 |
|  | 9 | 22.30772 | 22.15262 | 23.42858 | 34.46225 |
|  | 10 | 21.35393 | 20.16095 | 26.44332 | 20.87446 |
|  | 11 | 20.41111 | 15.94243 | 24.15365 | 30.77018 |
|  | 12 | 28.85403 | 31.73483 | 33.65518 | 31.575 |
|  | 13 | 19.26462 | 23.88802 | 33.55496 | 26.39707 |
|  | 14 | 20.75984 | 16.69308 | 23.27099 | 35.08104 |
|  | 15 | 32.1027 | 23.70774 | 26.24114 | 36.44343 |
|  | 16 | 23.77311 | 20.16576 | 23.2427 | 30.76636 |
|  | 17 | 23.83584 | 17.49818 | 18.51422 | 26.56248 |
|  | 18 | 25.29174 | 23.5833 | 24.54382 | 30.43965 |
|  | 19 | 23.17459 | 28.0383 | 14.50725 | 23.08064 |
|  | 20 | 22.04105 | 26.92183 | 30.39658 | 31.66242 |
|  | 21 | 21.07896 | 30.88544 | 31.73467 | 39.5222 |
|  | 22 | 20.74502 | 27.64956 | 25.18077 | 32.91746 |
|  | 23 | 27.6722 | 25.66307 | 20.63629 | 31.71266 |
|  | 24 | 26.74622 | 22.80893 | 20.70137 | 35.51939 |
